# The imPaCT study: a randomised controlled trial to evaluate a hospital palliative care team

**DOI:** 10.1038/sj.bjc.6600522

**Published:** 2002-09-23

**Authors:** G W Hanks, M Robbins, D Sharp, K Forbes, K Done, T J Peters, H Morgan, J Sykes, K Baxter, F Corfe, C Bidgood

**Affiliations:** Unit of Palliative Medicine, Division of Oncology, University of Bristol, Bristol Haematology and Oncology Centre, Horfield Road, Bristol BS2 8ED, UK; Division of Primary Health Care, University of Bristol, Cotham House, Cotham Hill, Bristol BS6 6JL, UK; Department of Social Medicine, University of Bristol, Canynge Hall, Whiteladies Road, Bristol BS8 2PR, UK; Bristol Haematology and Oncology Centre, Horfield Road, Bristol BS2 8ED, UK; Torbay Hospital, Lawes Bridge, Torquay, Devon TQ2 7AA, UK

**Keywords:** palliative care, programme evaluation, treatment outcome

## Abstract

A randomised controlled trial was undertaken to assess the effectiveness of a hospital Palliative Care Team (PCT) on physical symptoms and health-related quality of life (HRQoL); patient, family carer and primary care professional reported satisfaction with care; and health service resource use. The full package of advice and support provided by a multidisciplinary specialist PCT (‘full-PCT’) was compared with limited telephone advice (‘telephone-PCT’, the control group) in the setting of a teaching hospital trust in the SW of England. The trial recruited 261 out of 684 new inpatient referrals; 175 were allocated to ‘full-PCT’, 86 to ‘telephone-PCT’ (2 : 1 randomisation); with 191 (73%) being assessed at 1 week. There were highly significant improvements in symptoms, HRQoL, mood and ‘emotional bother’ in ‘full-PCT’ at 1 week, maintained over the 4-week follow-up. A smaller effect was seen in ‘telephone-PCT’; there were no significant differences between the groups. Satisfaction with care in both groups was high and there was no significant difference between them. These data reflect a high standard of care of patients dying of cancer and other chronic diseases in an acute hospital environment, but do not demonstrate a difference between the two models of service delivery of specialist palliative care.

*British Journal of Cancer* (2002) **87**, 733–739. doi:10.1038/sj.bjc.6600522 www.bjcancer.com

© 2002 Cancer Research UK

## 

Over the past 30 years in the UK there has been substantial expansion of palliative care services. Initially this was largely driven by the voluntary sector, but in recent years there has been not only endorsement by government but substantial investment. Specialist palliative care is now a required core service of every cancer network, and the UK government, in its recently published Cancer Plan (Department of Health, 2000), commits itself to improving equity of access to such services. Paradoxically, as specialist palliative care comes into the mainstream of health care, robust evidence of effectiveness is lacking. There are difficulties in producing such evidence which have been well rehearsed in recent years (Mcquay and Moore, 1994; Keeley, 1999). In particular, there have been few successfully completed randomised controlled trials (RCTs) (Rinck *et al*, 1997) and most of these have failed to demonstrate unequivocal benefits of specialist palliative care services when compared with usual care (Kane *et al*, 1985; Grande *et al*, 1999; Jourdhoy *et al*, 2000). Even where differences have been shown the gain has been modest. There have not, however, been any RCTs of hospital-based specialist palliative care. This is in spite of the fact that in the UK (and in several other countries) the biggest growth in palliative care services has been in the general hospital sector. Hospital-based ‘support’ teams are now widely established in district general hospitals and more services are being rolled out across the country. A report to the UK Department of Health in March 2000 revealed that 65% of acute hospitals had a specialist palliative care service (Clinical Standards Advisory Group, 2000), though these services varied considerably in their structure and available facilities.

We report here the first randomised controlled trial to evaluate a hospital specialist Palliative Care Team (PCT). The aim of the study was to compare outcomes (primarily symptom control, health-related quality of life (HRQoL), duration of hospital admission and rate of re-admission) in patients randomised to receive one of two models of advice and support from a multidisciplinary specialist PCT providing a package of care in addition to the usual care given in a university teaching hospital.

## METHODS

### Setting

The United Bristol Healthcare Trust (UBHT) comprises the Bristol Royal Infirmary with 433 beds and associated specialist hospitals providing services in oncology, ophthalmology, dentistry, otorhinolaryngology, paediatrics, and obstetrics and gynaecology. The oncology service sees nearly 4000 new patients a year. Within UBHT, the PCT has provided an advisory and consultancy service to hospital staff and patients since 1992. Any patient with palliative care needs can be referred to the PCT, with the consent of the patient's consultant. Patients with cancer or non-malignant illnesses are referred for symptom relief and pain control; emotional and psychological support for patients, carers and ward staff; patient discharge planning; social and financial advice; and bereavement support for carers. As well as providing a clinical service, the PCT undertakes a wide range of teaching including study days for nurses, provision of teaching modules within the undergraduate medical curriculum and postgraduate degree courses and supervision.

The study was approved by the UBHT local research ethics committee.

### Study population

All new inpatient referrals to the PCT were assessed for entry into the study. Initially only patients with cancer were included, but following a pilot study, all diagnostic groups were admitted, since non-cancer patients represent a significant proportion of the total (10%).

Referrals were received by a member of the PCT, who confirmed with the referring doctor or nurse that the patient was eligible for the study. Patients were excluded if they were unable to give informed consent, were not well enough to undertake the baseline assessment, were not aware of their diagnosis, were likely to die or be discharged within 24 h, or needed advice very urgently. Patients who expressed a strong preference to see the PCT or whose referring consultant ‘insisted’ that they be seen were also excluded. If the member of the PCT receiving the referral judged that the patient, their family or the ward staff were in ‘extreme distress’ the patient was excluded.

Eligible patients were visited by a researcher within 24 h of referral, to explain the study and obtain written informed consent. Patients were then randomised. The researchers who undertook the assessments were blind to the group allocation.

### The interventions

#### The full PCT service (‘full-PCT’)

This was the usual service delivered by the PCT, which during the study comprised two clinical academic consultants, one specialist registrar and three clinical nurse specialists (2.5 full-time equivalents). The PCT has close links with a clinical psychologist, a local hospice and community based palliative care services and access to social workers, rehabilitation staff and the chaplaincy in the hospital. Initial assessment of patients was undertaken by a specialist doctor or specialist nurse, either alone or together, and detailed advice about any problems identified was written in the patient's case notes and communicated to the patient's medical and nursing team personally or by telephone. Appropriate follow-up was then instituted which usually involved both telephone and in-person consultations with the patient, their family and the medical and nursing staff caring for the patient by one of the specialist nurses or the registrar. All patients were reviewed at least weekly by one of the consultants. For patients who were discharged from hospital, the PCT also provided liaison with community based health professionals and outpatient follow-up in the Palliative Care clinic if appropriate.

#### The control group (‘telephone-PCT’)

A more limited form of intervention was devised as a control. This involved no direct contact between the PCT and the patient or their family. Instead, within one working day of referral, a telephone consultation took place between a senior medical member of the PCT and the referring doctor and also between a PCT nurse specialist and a member of the ward nursing staff directly involved with the patient. A second telephone consultation could be made if necessary but thereafter no further follow-up or advice was given. Such a telephone advisory service commonly forms a part of the operational policy of specialist palliative care teams.

### Outcomes

The primary follow-up time was set at 1 week post-recruitment in order to maximise completeness of data. The four primary outcomes were: symptom control (including the severity of the most bothersome symptom identified by patients, mood, and emotional bother); HRQoL; and length of hospital stay and rate of re-admission. Secondary outcomes included satisfaction of patients, family carers and primary health care professionals; and use of health service resources.

#### Symptom control and HRQoL

HRQoL was measured by the EORTC QLQ-C30 questionnaire (items 29 and 30) (Aaronson *et al*, 1993); severity of most bothersome symptoms by visual analogue scales (VAS); mood by the Memorial Pain Assessment Card (MPAC) (Fishman *et al*, 1987); and extent to which emotional problems had been a bother by the WONCA scale (Scholten and van Weel, 1992). The measures of HRQoL and symptoms were repeated at weekly intervals for 4 weeks.

#### Hospital stay

The length of the index admission and rates of re-admission (until the patient's death or study closure) were recorded.

#### Satisfaction/dissatisfaction with care

Patient satisfaction with hospital care was assessed by four items derived from MacAdam's Assessment of Suffering Questionnaire (MacAdman and Smith, 1987). Patients were asked to nominate their primary home carer for the purposes of the study. The carer was sent a questionnaire within 3 days of the patient's recruitment which included the FAMCARE scale (Kristjanson, 1993), the Hospital Anxiety and Depression scale (HADS) (Zigmond and Snaith, 1983) and some additional questions about the way in which information and communication issues were handled in hospital. Carers were informed that they would be sent a similar questionnaire in the future. This questionnaire was timed to arrive 6 months after the death of the patient and consisted of the HADS and questions relating to the use of bereavement services.

A more detailed interview using a critical incident approach, was carried out with the carers of all patients who were discharged home. The interview covered satisfaction with the amount of information given about the patient's illness, medication, symptoms and sources of support; acceptability of current arrangements and location of care; expectations of future options for treatment and care and likely sequence of events.

The patient's GP and district nursing team were sent a letter following recruitment explaining the study and alerting the recipient that information would be requested at a later date about the patient's discharge arrangements. If a patient was not discharged from hospital no further contact was made with the primary health care team. For those patients who were discharged home, the GP and district nurse were interviewed by telephone, or in the case of repeated failure to arrange this, a questionnaire was sent. The questions covered satisfaction with the amount and type of communication with hospital staff; appropriateness of the package of care arranged for each patient; amount of input provided to the patient following discharge; and use of other community services by the patient.

#### Resource use data

Data on resource use in the hospital setting, by the PCT and in primary care were collected from a number of sources as detailed in Table 1

**Table 1:**
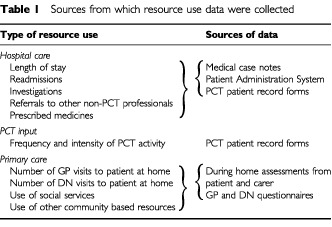
Sources from which resource use data were collected

.

#### Sample size

All new inpatient referrals to the PCT within UBHT were considered for entry to the study. A sample size of 261 patients was required in order to detect standardised differences in the four primary outcome variables of between 0.37 and 0.43 standard deviations with two-tailed α=0.05 and 80–90% power at the primary (1 week) follow-up using a 2 : 1 (intervention : control) randomisation ratio. It was considered that this would be a clinically important difference, would impact on patients and yet would be a feasible difference between the two groups in this setting.

### Randomisation

Randomisation took place after referral to the PCT: an unequal ratio was felt to be more acceptable to ward staff and was adopted in an attempt to facilitate recruitment. The randomisation was stratified by hospital site with patients from the Oncology Centre randomised separately from patients within the other hospitals of the group in order to ensure balance in the level of care received by patients in the study. In addition cancer and non-cancer patients were stratified within each hospital setting.

The randomisation schedule was prepared by generating random numbers on a computer (within Microsoft Access) in permuted blocks of three to ensure equality of randomisation between the strata. Randomisation details were recorded on adhesive labels placed in opaque non-resealable envelopes. Randomisation was undertaken by a non-clinical administrator with no involvement in patient recruitment or assessment.

### Data analysis

Established coding and data handling protocols relating to the use of structured instruments were followed. The randomised groups were compared on an intention to treat basis, including the use of confidence intervals. All analyses therefore included individuals in the group to which they were randomised, regardless of whether they subsequently switched groups. The first stage of the analysis used descriptive statistics to compare the groups at baseline in respect of socio-demographic characteristics and assessments at baseline. The primary analyses involved regression models comparing the allocated groups in respect of outcomes at follow-up, adjusting for baseline scores as covariates. The distributions of the outcomes together with the large sample sizes enabled parametric methods to be employed, and the median scores at baseline and follow-up were very similar to the means. All *P*-values presented are two-tailed.

Data from the semi-structured interviews with bereaved carers will be presented in a separate report.

## RESULTS

**Figure 1:**
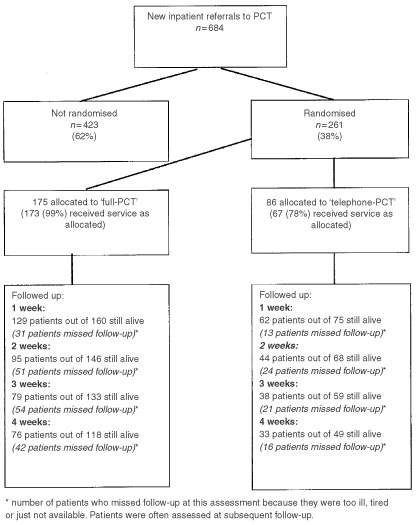
Flow of patients through the imPaCT trial

Every consultant in UBHT (apart from the obstetricians and paediatricians) agreed to allow patients under their care to be admitted to the study. With a view to entry into the study, 684 consecutive new inpatient referrals were assessed during the period July 1997 to April 2000 (Figure 1

). Of these, 261 (38%) were available for randomisation. Table 2

**Table 2:**
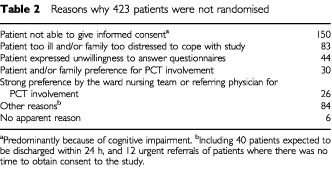
Reasons why 423 patients were not randomised

indicates the reasons why the remaining 423 patients were not available. These patients received usual care from the PCT (‘full-PCT’) unless they died or were discharged before contact could be made.

Table 3

**Table 3:**
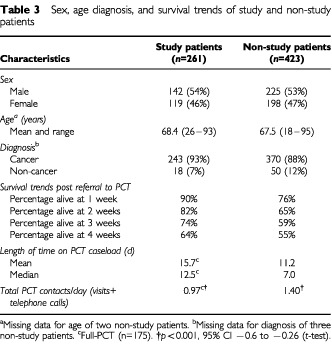
Sex, age diagnosis, and survival trends of study and non-study patients

compares the baseline characteristics of patients who agreed to participate in the study with those who either refused or were not suitable for inclusion. The two populations did not differ markedly with respect to age, sex and diagnosis or primary cancer site. However, the non-study population was more ill and closer to death. For patients with a known date of death the mean length of survival for study patients was 76.3 days post-referral to the PCT (median 33 days) compared with 61.5 days for non-study patients (median 23 days).

There were also differences between the study patients randomised to ‘full-PCT’ and non-study patients in the length of time on caseload and intensity of input from the PCT (Table 3). The PCT had more contacts per day with non-study patients and the difference was statistically significant.

### Baseline characteristics

The allocated groups were similar in baseline characteristics except in gender distribution (Table 4

**Table 4:**
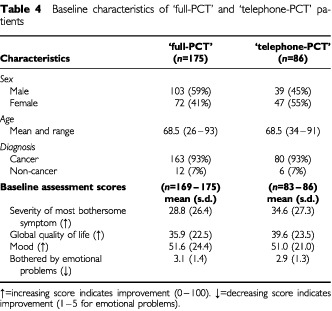
Baseline characteristics of ‘full-PCT’ and ‘telephone-PCT’ patients

). The prevalence of the most bothersome symptom volunteered by patients at the baseline assessment was also similar in the two allocated groups (Table 5

**Table 5:**
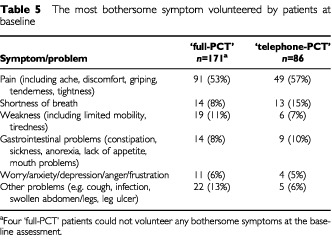
The most bothersome symptom volunteered by patients at baseline

).

### Primary outcomes

Table 6

**Table 6:**
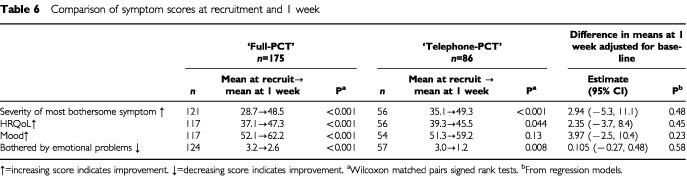
Comparison of symptom scores at recruitment and 1 week

shows the change in scores within allocated groups from baseline to the week 1 assessment. These data show that there was a highly significant improvement in scores for all items in the ‘full-PCT’ group and for some items in the ‘telephone-PCT’ group. However, comparison of the mean scores at 1 week adjusted for the baseline scores revealed no statistically significant differences between the groups. The improvements in scores for symptom severity, mood, emotional problems and HRQoL, which were apparent at 1 week, were, amongst survivors, sustained and increased over the subsequent 3 weeks (Figure 2

**Figure 2:**
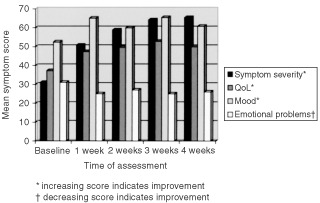
Weekly mean symptom scores of those patients who completed all six assessments (*n*=76)

).

There was very little difference in the length of hospital stay or rates of readmission between the two groups: length of stay 14.7 (9.4) (mean (s.d.)) days ‘full-PCT’; 13.2 (9.6) days ‘telephone-PCT’; 0.18 (0.4) readmissions ‘full-PCT’; 0.18 (0.4) readmissions ‘telephone-PCT’.

### Secondary outcomes

#### Patient and carer satisfaction/dissatisfaction

Patients in both treatment groups expressed high levels of satisfaction with their hospital care and there were no apparent differences between the groups (Table 7a

**Table 7a:**
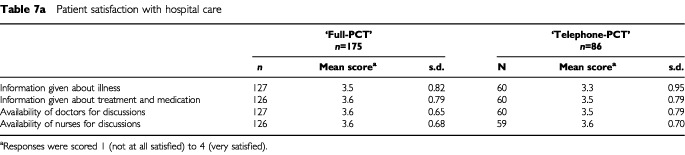
Patient satisfaction with hospital care

,bTable 7b

**Table 7b:**
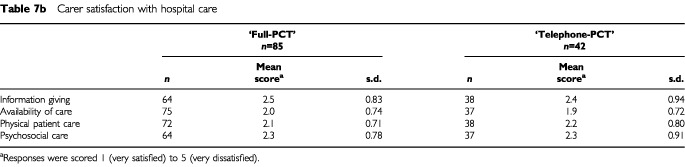
Carer satisfaction with hospital care

).

#### Hospital resource use

Hospital resource use (number of diagnostic images, diagnostic tests, visits from other hospital therapists) was very similar in the two groups (data not shown).

#### Utilisation of GP and district nurse services

One hundred and twenty-five study patients (48%) were discharged from hospital to their home within the study period. Patients who had been allocated to ‘full-PCT’ spent on average fewer days at home than patients in the ‘telephone-PCT’ group: 13.2 (8.1) (mean (s.d.)) ‘full-PCT’; 15.9 (7.9) ‘telephone-PCT’. However, these patients also received more GP visits per day spent at home and this was statistically significant: 0.23 (2.3) ‘full-PCT’; 0.13 (0.13) ‘telephone-PCT’, *P*<0.01 (*t*-test). Patients who received ‘full-PCT’ also received more district nurse visits but this was not statistically significant: 0.45 (0.59) ‘full-PCT’; 0.34 (0.54) ‘telephone-PCT’.

#### GP and district nurse satisfaction

GPs and district nurses were asked about the adequacy of the notice given of the patient's discharge, whether a shared care form had been received by the district nurses, and overall satisfaction with the discharge procedure (timing, information, equipment planning). These data revealed a tendency for the primary care professionals of patients who received ‘full-PCT’ to be more satisfied with discharge arrangements than those of patients in the ‘telephone-PCT’ group, but this difference did not achieve statistical significance.

### Secondary analyses

An explanatory analysis was carried out to explore the effect of proximity to death on symptom score differences. Length of survival from referral to the PCT to date of death or date of study closure (30 April 2000) was calculated and the population of study patients divided according to whether death occurred within 28 days of referral or not. The difference between the randomised groups in symptom severity at one week was not influenced by proximity to death.

#### Non receipt of allocated intervention

Nineteen patients switched from ‘telephone-PCT’ to ‘full-PCT’ during the course of the study (10 within the first week) and two patients switched in the other direction (both in the first week). The decision to switch intervention groups was only taken after consultation between the patient's referring team and senior medical PCT staff and was discouraged as far as possible. When a patient did switch groups it was usually because the ward staff felt that they needed more help with the patient's care, particularly symptom control and psychosocial support. The switch to ‘telephone-PCT’ was on both occasions because the ward staff realised that the patient had not been prepared for face-to-face PCT input. In these two cases telephone advice was regarded as more appropriate than visits (and neither patient was ever seen by the PCT).

## DISCUSSION

This is the only randomised controlled trial of a specialist hospital advisory PCT. It has failed to show a significant difference between the ‘full-PCT’ and ‘telephone-PCT’ in respect of the primary outcome measures, and particularly symptoms and HRQoL. However, we have completed meticulously a randomised controlled trial that provides robust, detailed and comprehensive information about the clinical course and care of patients dying of cancer and other chronic diseases in an acute hospital setting. We are able to conclude from our data that in both treatment groups the management of patients entered into the study (as reflected in the primary outcome measures) was of a high standard, that their symptoms were significantly improved, that patients and carers were highly satisfied with the care they received and the information they were given, and that communication and liaison between the hospital and primary care teams was good and generally of a high order. These are important findings, irrespective of the comparison of interventions.

In both groups there were highly significant improvements in bothersome symptoms and HRQoL which were apparent within 1 week and sustained for the duration of the 4 week observation period. The usual course in such patients is one of deterioration in physical symptoms (Coyle *et al*, 1990; Ventafridda *et al*, 1990, Peruselli *et al*, 1999). There was a consistent direction to the comparisons of the main outcome measures and satisfaction scores in favour of ‘full-PCT’, though the differences between the groups were not significant. However, there was a highly significant difference in the number of patients who switched intervention groups, in favour of the ‘full-PCT’. It seems clear from these data that patients in both groups were being well managed. This poses the question as to whether either or both interventions contributed to this good management.

Early studies completed before the development of hospice and specialist palliative care showed that physical and psychological symptoms were common and often inadequately treated (Hinton, 1963, 1964). Specialist services for dying patients began in the 1960s and 1970s, but the quality of care in hospitals and the community inevitably varied in different localities. In one survey published in 1984 of a random sample of 262 deaths, ‘a high proportion’ had ineffectively controlled pain, cough, dyspnoea or insomnia (Wilkes, 1984).

A large survey of family members of people who died from cancer in the UK in 1990 indicated that pain and other symptoms in these patients were common and caused considerable distress (Addington-Hall and Mccarthy, 1995). Sixty-one per cent of patients experienced pain in their last week of life and other symptoms (dyspnoea, vomiting and constipation) were common and were often not well controlled. The same study highlighted deficiencies in giving information to and communication with patients and their families. More recently, the SUPPORT study in five teaching hospitals in the United States surveyed more than 4000 patients hospitalised with life-threatening diagnoses. The data highlighted major problems of unrelieved pain and failures of communication (The Support Principal Investigators, 1995). For example, for 50% of the conscious patients who died in hospital, family members reported moderate or severe pain at least half of the time. The findings in these studies have been reflected in emotive and sometimes harrowing case reports (Anonymous, 1989, 1994; Fenton, 1992) which have drawn attention to widespread failures in management of patients dying in acute hospitals. In contrast, the positive changes we have demonstrated in the primary outcome measures in this study suggest that patients received a high standard of care in both groups.

The lack of a significant difference between the two interventions may be a false negative in that there is a difference but we have failed to show it. There are some pointers in our findings which indicate that this may be the case. The factors which may have contributed include the selection of an unrepresentative sample of patients; contamination of the control group; and insufficient numbers of patients. In the planning of this study we had anticipated all of these potential difficulties of conducting an RCT which have been highlighted by others (Kane *et al*, 1985; Mcwhinney *et al*, 1994; Aranda, 2000) and had taken steps to overcome them (the unequal randomisation ratio, broad entry criteria, and maintenance of a high level of awareness of the study amongst all hospital staff over a 2-year period). In spite of this only 38% of potential patient recruits were randomised. Our data indicate that the non-randomised patients were less well and had a shorter survival time. Clinical activity data show that they received more intensive input from the PCT than either study group. It seems that we have excluded from the study a group of patients who may be most needy in terms of specialist palliative care and who may have been more likely to show differences between the interventions. One of the major challenges in palliative care research is to develop measures to capture outcome data from such patients in a way that is practical and ethical.

The best design for this RCT would have been a comparison of ‘full-PCT’ with no input from the PCT at all. This was rejected on ethical grounds on the basis that randomisation would take place after referral to the PCT. Instead we decided to provide a minimal telephone service intervention as the ‘control’, but we probably allowed this telephone intervention to develop too far into a structured package of care in its own right.

Another factor which is likely to have influenced the care of patients in the control group was the educational activity of the PCT within the hospital which continued as normal throughout the study. Regular seminars were held for oncology house staff (who often then moved into the main hospital) and study days were organised for nursing staff throughout the hospital. In addition the day-to-day activity of the PCT within the hospital would have had a significant educational influence.

The aim of the study was ambitious: to demonstrate a difference in outcomes (if one exists) between two levels of advice and support from a multidisciplinary PCT providing a package of care in addition to the usual care given in a university teaching hospital. In the light of our ‘negative’ result questions will be asked about our trial design and in particular whether a randomised controlled trial was the best option. We believe a RCT was the right design to use. By imposing the rigour necessary to undertake such a study, we have collected high quality and comprehensive data. It is not the research paradigm which has failed, rather it is the inadequacy of the measures in capturing outcome data from patients who are very ill and frail.

In conclusion, we were not able to differentiate clearly in an acute hospital setting between two models of service delivery of specialist palliative care, partly because both were associated with highly significant improvements in the primary outcome measures. Our study shows a high level of care for patients dying in this setting in this particular group of hospitals. We believe that this reflects the fact that there have been major improvements in the last two to three decades in the way that dying patients are looked after. These have not been implemented universally but the activity of a specialist PCT in a general hospital is likely to raise standards of care for such patients. We agree with Wilkes (1993) that ‘we can look back and see that the hospice movement has irreversibly improved the standards of care for the dying’. Our data also demonstrate that this quality of care can be effectively provided to patients in acute hospital beds.
